# Clinical and environmental considerations for neonatal, office-based circumcisions compared with operative circumcisions

**DOI:** 10.3389/fruro.2024.1380154

**Published:** 2024-07-03

**Authors:** Benjamin Press, Michael Jalfon, Daniel Solomon, Adam Benjamin Hittelman

**Affiliations:** ^1^ Yale School of Medicine, Department of Urology, School of Medicine, Yale University, New Haven, CT, United States; ^2^ Department of Surgery, Division of Pediatric Surgery, Yale School of Medicine, New Haven, CT, United States

**Keywords:** circumcision, office based procedures, environmental footprint, healthcare disparities, operating room

## Abstract

Neonatal male circumcision is a commonly performed procedure in the United States. Circumcisions are performed at various ages by a variety of clinical providers for multiple reasons, including religious, cultural, personal, and medical indications. In the United States, neonatal circumcision is routinely performed by non-urologic providers in the hospital within the first few days of life or as a religious ceremony on the 8^th^ day of life. If neonatal circumcision is deferred in the hospital and subsequently not performed in the outpatient setting, it is then typically performed in the operating room under general anesthesia after 6 months of life. Neonatal circumcision is supported by both the American Academy of Pediatrics (AAP) and the American College of Obstetricians and Gynecologists (ACOG) due to the belief that the health benefits outweigh the minimal risk of the procedure. Despite this, neonatal circumcision rates have decreased in the United States in recent decades, in part due to access to the procedure, often related to changing insurance coverage. This has led to increased rates of operative circumcisions. Operative circumcisions are more costly to the healthcare system, subject the patient to cardiopulmonary and potentially neurotoxic effects of general anesthesia, and carry an increased environmental footprint, compared to neonatal circumcision. The intention of this paper is not to promote or justify circumcision for all patients, but rather to compare the clinical and environmental impact of neonatal versus operative circumcisions.

## Introduction

Neonatal male circumcision is widely performed in the United States. Over 50% of boys in the United States undergo circumcision (1, 2). Circumcision can be performed at different ages by a variety of providers and performed for religious, cultural, personal, or medical indications. In the United States, neonatal circumcision is routinely performed by non-urologic providers in the hospital within the first few days of life. Traditional Jewish circumcisions are performed on the 8^th^ day of life in the home. If neonatal circumcision is deferred, the procedure is then commonly performed by a Pediatrician, Family Practice Physician or Pediatric Urologist within the first few weeks to month of life in the outpatient clinic. Neonatal circumcisions are generally safe and well tolerated, with rates of minor complications (bleeding, infection) of approximately 1–2% and major complications (amputation of glans, urethral injury, need for re-operation) of < 1% (3). While neonatal circumcisions are commonly performed under local anesthetic, neonatal circumcisions that are deferred in infancy are then typically performed in the operating room under general anesthesia after 6 months of life, though some centers are beginning to offer the procedures under spinal anesthetic.

Based on a systematic review of the English literature, the American Academy of Pediatrics (AAP) endorsed that the health benefits of newborn male circumcision outweigh the risks of the procedure (4). Thus, the AAP promotes that circumcision should be covered by third party insurance providers. The American College of Obstetricians and Gynecologist also supported this statement (4). This statement is also in line with that of the American Urologic Association, citing the prevention of phimosis, paraphimosis and balanoposthitis, reduced communicable sexually transmitted diseases and HIV, as well as markedly decreased incidence of penile cancer (5). In addition, there is a 90% reduction of urinary tract infections in circumcised *vs*. uncircumcised boys during the first three to six months of life (6). However, despite advocacy for neonatal circumcisions, there remains discrepancies in the access for circumcision in newborn males. The downstream effects are felt by the patient, health system, and also the environment. This article will herein summarize these downstream impacts.

## Costs and access

Boys who were not circumcised during the neonatal period due to prematurity, serious illness, or concerns about genital abnormalities are frequently deferred and referred to Pediatric Urologists to have the procedure performed. If it is not completed in the neonatal period, the circumcision is then deferred to operating room under general anesthesia or spinal anesthetic. While it is well known that the cost of performing a procedure in the operating room carries significant increased expense when compared to the procedure done in the ambulatory setting, the true scale of the cost differences may not be common knowledge among practitioners. Jayanti et al. evaluated the cost of ambulatory *vs*. operative circumcision over a 30 month period. While the average circumcision performed in the office cost $196 for facility and equipment, circumcisions in the operating room averaged $1,805 for the facility, equipment, and anesthesia costs. By performing circumcision with local anesthesia in an office setting, the group was able to save $184, 713 annually over the course of a 30 month period (7). These cost discrepancies were corroborated by Many et al., who found that the median cost of an operative circumcision was $2892, whereas the median cost of neonatal circumcision was approximately $173 (8).

Despite the purported benefits of circumcision itself, as well as the cost savings and medical benefit of avoiding general anesthesia, access to neonatal circumcision has been limited in certain communities. Dr. Emilie Johnson and the research group from Northwestern performed a retrospective review of circumcision in over 8 million boys in the Kids’ Inpatient Database between 2003 and 2016. During that period, the rates of neonatal circumcision decreased from 57.4% to 52.1%, while the rates before and after the 2012 AAP statement advocating to increase access to circumcision did not differ statistically (1). The authors found that there were geographical and income related disparities in neonatal circumcision rates. In general, neonatal circumcision rates increased relative to higher income, with 50.3% of boys in the lowest income quartile undergoing neonatal circumcision compared to 60.7% of boys in the highest income quartile. In addition, privately insured boys (64.9%) underwent neonatal circumcision at a higher rate than publicly insured boys (44.6%) (1). A subsequent review of the pediatric health information system (PHIS) database found that in states where Medicaid does not cover newborn circumcisions, there were higher rates of operative circumcision, despite similar rates of phimosis diagnoses. Furthermore, there was a greater than two-fold increase in balanitis cases in states in which Medicaid did not cover neonatal circumcisions (9). In Colorado and Florida, following defunding for neonatal circumcision, rates of neonatal circumcision were reduced by 20.9% and 16.0%, respectively. Black neonates appeared to be disproportionately impacted by changes in Medicaid coverage compared to their white counterparts. When Medicaid coverage was available, black neonates had higher rates of circumcision than white neonates. When Medicaid coverage was unavailable, white neonates were circumcised at a higher rate than black neonates (10).

## Anesthesia risk and complications

Surgery on a pediatric patient typically requires the use of general anesthesia, in contrast to the adult population, where regional anesthesia may be used as an alternative to general anesthesia. While regional anesthesia is frequently combined with general anesthesia to lessen perioperative pain and anxiety, there are risks associated with anesthesia in young patients. Recent research has brought attention to these risks by indicating that repeated or prolonged exposure to anesthesia during early childhood may have a negative impact on brain development (11, 12).

Neuronal cell death, impaired neurogenesis, glial death, and abnormal axon formation have been shown to occur in animal models after exposure to general anesthetics at a young age (13–15). Other models have also found that anesthesia exposure in infancy is associated with altered behaviors including heightened emotional reactivity to threats, and impaired learning and memory formation persisting into early adulthood (16, 17).

In 2016, the U.S. Food and Drug Administration warned “repeated or lengthy use of general anesthetic and sedation drugs during surgeries or procedures in children younger than 3 years or in pregnant women during their third trimester may affect the development of children’s brains.” (18) This warning was made based on review of largely animal studies as well as a few human studies. Despite this, the evidence regarding neurocognitive outcomes in human studies is conflicting, with significant heterogeneity in populations and outcome measures (19). A more recent randomized control trial, found that neurodevelopment after just under an hour of general anesthesia in early infancy does not significantly differ when compared to awake-regional anesthesia (20). While perioperative mortality in non-cardiac surgeries has been reported as only 1.6 per 10,000 anesthetics (21), there still poses a risk of cardiopulmonary events including bradycardia/hypotension, laryngospasm, aspiration, and apnea (22).

For non-financial reasons, circumcision under local anesthesia has the advantage of mitigating the potential associated risks of general anesthesia. Parents will often prefer neonatal circumcision to avoid the potential associated risks of general anesthesia. More time is devoted to operative procedure as patients will spend time in the preoperative holding areas, anesthesia consultation, and recovery room. The procedure takes less time in an office setting, and the child can typically return home faster.

## Environmental footprint

While our knowledge of how climate change affects human health has grown recently, less is known about how health care services negatively impact the environment. The United States is one of the world’s leaders in greenhouse gas emissions. From 2003–2013 greenhouse gas emissions from the nation’s healthcare system increased by 30%, to account for nearly 10% of the nation’s total greenhouse gas emissions (23). The U.S. healthcare system has been shown to be a significant contributor to air pollution, accounting for a significant percentage of the downstream effects of carbon emissions. This includes acidification (12%), smog formation (10%) and respiratory disease from particulate matter (9%), as well as contribution to ozone depletion and carcinogenic and non-carcinogenic air toxins (1–2%) (23). Current research has focused on the environmental impact of products and processes with the hospital system. Life cycle assessments have been performed to quantify the environmental and public health impact of such processes in the hospital system, such as the production, use and disposal of medical equipment. Dr. Jodi Sherman, has been one of the foremost researchers in this area, evaluating opportunities for reductions in emissions, including but not limited to the decreased use of single use laryngoscopes (24), disposable materials and single-use surgical devices in hysterectomies (25), and even reducing unnecessary prostate biopsies (26).

At our own institution, we sought to evaluate the environmental impact of office-based clamp neonatal circumcision *vs* operative circumcision by measuring the weight of disposable materials as a surrogate. Average weights of disposable materials were significantly higher for operative circumcisions than for clinic circumcisions (3.71 ± 0.73 kg *vs*. 0.38 ± 0.11 kg, p < 0.001). The weight of disposable materials for circumcisions performed in the operating is nearly 10 fold higher than in the clinic setting. The environmental and financial impact of the added waste must be considered when planning for these procedures. Early referral to Pediatric Urologists in the newborn period could potentially allow for increased access to non-operative newborn circumcisions in the clinic in the first few weeks to months of life.

There is potential for redundant skin or poor cosmetic outcome after a neonatal circumcision. In this case, families may elect to have the circumcision revised in the operating room. Over the last 12 years at out institution, only 933 revisions of circumcisions have been performed while there were 39,914 neonatal circumcisions performed within that same time period. Even if we account for the potential for poor cosmetic outcome and anticipation of revision of circumcision, the numbers do not justify deferral to perform circumcision in the operating room.

## Discussion

Neonatal circumcision is performed for a variety of clinical, personal and religious indications. While we are not promoting circumcision as a practice for all patients, we recognize the medical and personal benefits and advocate that it should be available for families who choose the procedure. However, due to changes in access for neonatal circumcision, we are observing declining rates of neonatal circumcision over time (1). Many parents desire circumcision for their children, as evidenced by the increase in operative circumcisions in states that do not cover neonatal circumcisions under Medicaid (9). While operative circumcisions are generally safe and well tolerated by the patient, they are a more complex and involved procedures than neonatal circumcisions performed in the hospital or clinic, with increased costs and environmental impact. Operative circumcisions require general anesthesia which introduces additional cardiopulmonary risks to the child, as well as potentially neurotoxicity to the child’s developing brain. Operative circumcisions also have higher rates of complications compared to neonatal circumcision (27).

Lack of access to neonatal circumcision typically reflects insurance coverage and low insurance reimbursement (28). This is in contrast to the AAP Policy Statement, which explicitly states that “the benefits of circumcision are sufficient to justify access to this procedure for families choosing it and to warrant third-party payment for circumcision of male newborns” (4). Training by non-urologic providers for neonatal circumcision is becoming less ubiquitous, possibly due to the poor reimbursement (29). A review of program procedural requirements for obstetrics and gynecology residency do not include circumcision (30, 31). To alleviate this potential need for practitioners of neonatal circumcision, there has been increased utilization of advanced practice providers, a practice that has been deemed to be both feasible and safe (32). However, without improved coverage and better reimbursement, there will likely be continued deferment of circumcision from the neonatal period to later in life.

Further research needs to be done to further quantify the healthcare costs associated with variable access to neonatal circumcision, both fiscally, and environmentally. As environmental stewards, we should be cognizant of the environmental footprint of the most prevalent procedure in Pediatric Urology.

## Author contributions

BP: Writing – original draft, Writing – review & editing. MJ: Writing – review & editing. DS: Writing – review & editing. AH: Writing – original draft, Writing – review & editing.
